# Occupational Physical Stress Is Negatively Associated With Hippocampal Volume and Memory in Older Adults

**DOI:** 10.3389/fnhum.2020.00266

**Published:** 2020-07-15

**Authors:** Agnieszka Z. Burzynska, Daniel C. Ganster, Jason Fanning, Elizabeth A. Salerno, Neha P. Gothe, Michelle W. Voss, Edward McAuley, Arthur F. Kramer

**Affiliations:** ^1^Department of Human Development and Family Studies, Molecular, Cellular and Integrative Neurosciences Graduate Interdisciplinary Studies Program, Colorado State University, Fort Collins, CO, United States; ^2^Department of Management, Colorado State University, Fort Collins, CO, United States; ^3^Department of Health & Exercise Sciences, Wake Forest University, Winston-Salem, NC, United States; ^4^Cancer Prevention Fellowship Program, Division of Cancer Epidemiology & Genetics, National Cancer Institute, Bethesda, MD, United States; ^5^Department of Kinesiology and Community Health, University of Illinois at Urbana–Champaign, Urbana, IL, United States; ^6^Department of Psychological and Brain Sciences, University of Iowa, Iowa City, IA, United States; ^7^Beckman Institute for Advanced Science & Technology, University of Illinois at Urbana–Champaign, Urbana, IL, United States; ^8^Department of Psychology, Northeastern University, Boston, MA, United States; ^9^Department of Mechanical and Industrial Engineering, Northeastern University, Boston, MA, United States

**Keywords:** aging, hippocampus, occupation, stress, complexity, memory

## Abstract

Our jobs can provide intellectually and socially enriched environments but also be the source of major psychological and physical stressors. As the average full-time worker spends >8 h at work per weekday and remains in the workforce for about 40 years, occupational experiences must be important factors in cognitive and brain aging. Therefore, we studied whether occupational complexity and stress are associated with hippocampal volume and cognitive ability in 99 cognitively normal older adults. We estimated occupational complexity, physical stress, and psychological stress using the Work Design Questionnaire (Morgeson and Humphrey, 2006), Quantitative Workload Inventory and Interpersonal Conflict at Work Scale (Spector and Jex, 1998). We found that physical stress, comprising physical demands and work conditions, was associated with smaller hippocampal volume and poorer memory performance. These associations were independent of age, gender, brain size, socioeconomic factors (education, income, and job title), duration of the job, employment status, leisure physical activity and general stress. This suggests that physical demands at work and leisure physical activity may have largely independent and opposite effects on brain and cognitive health. Our findings highlight the importance of considering midlife occupational experiences, such as work physical stress, in understanding individual trajectories of cognitive and brain aging.

## Introduction

An average full time worker in the United States spends 8.56 h at work per weekday (Bureau of Labor Statistics, 2016) and remains in the workforce for about 40 years (Komp-Leukkunen, 2019). Thus, occupational experiences likely play an important role in cognitive and brain aging. On the one hand, a job can provide an intellectually and socially enriched environment, supporting cognitive function. Indeed, midlife occupational complexity with people and data has been associated with better verbal skills, memory, and speed of processing after retirement (Finkel et al., 2009; Smart et al., 2014; Andel et al., 2015, 2016). On the other hand, work is the second main source of stress among employees (American Psychological Association, 2015). Both psychological and physical stress (e.g., physical hazards) at work have been linked with poorer cognitive ability in midlife (McEwen and Sapolsky, 1995; Sandström et al., 2005; Scott et al., 2015) and after retirement (Gow et al., 2014; Andel et al., 2015; Sindi et al., 2017; Dong et al., 2018).

To better understand the neural underpinnings of the above associations, we recently proposed the “Brain Aging: Occupational Stimulation and Stress” (BOSS) model (Burzynska et al., 2019). The BOSS model acknowledges the possibly opposing influences of occupational complexity and stress on cognitive aging. Specifically, it posits that occupational enrichment may protect against age-related cognitive decline, either by supporting cognitive reserve [i.e., providing mind’s resistance to biological brain aging (Stern et al., 2018)] or by supporting brain maintenance [i.e., minimizing age-related neural losses (Nyberg, 2017)]. Conversely, occupational stress may deplete the brain reserve and accelerate age-related changes through a variety of neural and systemic processes (Burzynska et al., 2019).

The hippocampus is the ideal first target for studying the BOSS model. First, the hippocampus undergoes a reduction in volume in both healthy aging (Scahill et al., 2003; Kennedy and Raz, 2005) and dementia (Barnes et al., 2009), which is associated with declines in memory and general cognition (Van Petten et al., 2004; Den Heijer et al., 2010; Gorbach et al., 2017). More importantly, the hippocampus is the brain structure where occupational stimulation and stress may converge. For example, research on both humans and animals shows that enriched environments promote hippocampal neurogenesis, neuroplasticity, and neurotrophic support (Bettio et al., 2017). Alternatively, psychological and physical stress inhibits hippocampal neurogenesis, neurotrophic support, and can induce neurotoxicity via upregulation of glucocorticoid cascade, inflammation, or oxidative stress (Nagata et al., 2009; Choi et al., 2014; Bettio et al., 2017; Burzynska et al., 2019).

Currently, the evidence for the effects of occupational stimulation and stress on the hippocampus is scarce. Greater midlife managerial experience has been associated with greater hippocampal volume and lesser atrophy over a period of 2.5 years in older adults (Suo et al., 2012, 2017), and taxi drivers had greater hippocampi than bus drivers, where hippocampal volume correlated with their navigating experience (Maguire et al., 2006). This suggests that certain types of occupational complexity may support brain health maintenance. Conversely, another study demonstrated that greater work complexity with data, people, and things, when controlling for cognitive function, was associated with smaller hippocampi in middle-aged adults at risk of Alzheimer’s Disease (Boots et al., 2015) this lends support to the cognitive reserve theory. Finally, others found no association between early-life occupational complexity [estimated from the O^∗^NET occupational codes; (Kaup et al., 2018)] or exposure to novelty [i.e., work task changes (Oltmanns et al., 2017)] and hippocampal volume in middle age. To our knowledge, the one existing study linking occupational stress to brain structure found that burnout in middle-aged workers was related to decreased volumes of several brain regions, but not the hippocampus (Blix et al., 2013).

In sum, the associations between occupational complexity, stress, and hippocampal health in older age need to be further investigated to understand individual differences in cognitive and brain aging. Therefore, the current study related occupational characteristics to hippocampal volume and cognitive function in healthy aging. The unique aspect of our study was the direct and subjective (as opposed to derived from occupational codes) assessment of stimulating and stressful work characteristics using three validated questionnaires. We hypothesized that older adults who experienced more cognitive complexity at work would have larger hippocampi and better cognition, and those who reported more occupational stress (either physical or psychological) would have smaller hippocampi and poorer cognition, after controlling for age, gender, and brain size. Importantly, our key question was whether occupational experiences are related to hippocampal volume and cognition beyond the known proxies of cognitive reserve related to socioeconomic status (e.g., education, occupational attainment, and income), as well as general stress and leisure physical activity.

## Methods

### Participants

The current study was conducted using the MRI, cognitive, and other baseline data from a 6-month randomized controlled exercise trial (clinical study identifier NCT01472744). Healthy, low active older adults were recruited in Champaign County in Illinois. Of the 1,119 participants recruited, 247 (*n* = 169 women, *n* = 78 men) met inclusion criteria of the clinical trial, agreed to enroll in the study, and underwent a series of demographic, health, neuroimaging, cognitive, and cardiorespiratory data collection at baseline. For more details on this clinical trial, its primary outcomes and neuroimaging data, refer to our earlier work (Burzynska et al., 2017; Ehlers et al., 2017; Fanning et al., 2017; Baniqued et al., 2018; Voss et al., 2019). Eligible participants met the following criteria to be enrolled in the clinical trial: (1) were between the ages of 60 and 80 years old; (2) were free from psychiatric and neurological illness and had no history of stroke, transient ischemic attack, or head trauma; (3) scored ≥23 on the Mini-Mental State Exam (MMSE) and >21 on a Telephone Interview of Cognitive Status (TICS-M) questionnaire; (4) scored < 10 on the geriatric depression scale (GDS-15); (5) scored ≥75% right-handedness on the Edinburgh Handedness Questionnaire; (6) demonstrated normal or corrected-to-normal vision of at least 20/40 and no color blindness; (7) were screened for safe participation in an MRI environment (e.g., no metallic implants that could interfere with the magnetic field or cause injury and no claustrophobia); and (8) reported to have participated in no more than two moderate bouts of exercise per week within the past 6-months. The baseline data were collected in four waves in years 2011–2014.

In 2017 we sent questionnaires assessing occupational characteristics to participants who indicated interest in follow-up assessments; 100 participants returned the completed work questionnaire and participants with MMSE ≤ 23 were excluded from final analyses due to possible mild cognitive impairment (Marioni et al., 2011). Seventy of 99 participants were women, 76 identified as Caucasian White, six as Black, one as Asian, and 16 decided not to answer; 83 identified as Non-Hispanic 16 decided not to respond; 96 had MRI data (Table 1).

**TABLE 1:** Sample characteristics.

	*N*	Min	Max	*M*	*SD*
Age (at MRI)	99	60	79	65.3	3.1
Education (years)	99	12	26	16.3	3.1
MMSE	99	26	30	28.7	1.3
eTIV (mm^3^)	96	1205091	2054710	1457556	151131
Hippocampal volume (mm^3^)	96	6484	10140	8319	848
Years in current occupation	99	1.5	51.5	18.4	13.4
Years MRI/cog – occupation	99	3	9	4.6	1.1
PASE leisure score	99	5	254	114	46
Stress (PSS-10)	99	0	28	12	6

To estimate cognitive reserve socioeconomic factors, we used years of education, income and job title collected at the clinical trial baseline. The participants were asked to choose the following household income categories: (1) <$5,000, (2) $5,001–10,000, (3) $10,001–$15,000, (4) 15,001–20,000, (5) 20,001–25,000, (6) 25,001–30,000, (7) 30,001–40,000, (8) >40,000. Sixty-five subjects responded >40,000, six 30,000–40,000, two 25,001–30,000, three 20,001–25,000, three 15,001–20,000, and four 10,000–15,000; 16 chose not to respond. The number of participants who identified with the following job titles were: (1) officials and managers (*n* = 26), (2) professionals (*n* = 29), (3) technicians (*n* = 3), (4) sales (*n* = 6), (5) office and clerical (*n* = 14), (6) craft workers (skilled, *n* = 3), (7) operatives (semiskilled, *n* = 10), (8) laborers (unskilled, *n* = 1), (9) service workers (*n* = 6), (10) homemakers (*n* = 1). In addition, we asked the participants about their current employment status: 13 worked full time, 10 part time, 26 were retired but employed, and were retired and not employed.

### MRI Acquisition

Structural images were acquired on a 3 T Siemens Trio Tim system (Siemens, Erlangen, Germany) using a T1-weighted 0.9 mm^3^ MPRAGE sequence (TR = 1,900 ms, TE = 2.32 ms, TI: 900 ms, FA = 9°; matrix = 256 × 256; FOV = 230 mm; 192 slices; GRAPPA acceleration factor 2).

### MRI Data Processing

Automated brain tissue segmentation and reconstruction of cortical models was performed on T1-weighted images using the Freesurfer software, version 5.3^1^. Individual T1-weighted images underwent non-brain tissue removal, Talairach transformation, and creation of representations of the gray/white matter boundaries (Dale and Sereno, 1993; Fischl et al., 1999). AZB screened all reconstructions to evaluate the success and plausibility of the automatically processed results, as recommended by the software developers. Volumes of estimated total intracranial volume (eTIV), left and right hippocampus were extracted per individual; total volume of hippocampus was calculated as the sum of bilateral volumes (Table 1).

### Subjective Occupational Experiences

Participants were instructed to answer the questionnaires with regard to their most recent job that they had been performing (full time or part-time) for 2 years or longer. We focused on the most recent job to minimize the effects of age-related declines in memory (Park and Festini, 2017). Subjective occupational experiences were assessed with the 77-item Work Design Questionnaire (WDQ) that results in 21 occupational factors (Morgeson and Humphrey, 2006), the 5-item Interpersonal Conflict at Work Scale (Spector and Jex, 1998), and the 4-item Quantitative Workload Inventory (Spector and Jex, 1998). Both have been validated and tested in different contexts (Bayona et al., 2015; Wright et al., 2017). Among the total of 23 factors across the three questionnaires, we identified six factors that refer to cognitive job complexity (task variety, job complexity, information processing, problem solving, skill variety, and specialization), two related to psychological stress (workload and interpersonal conflict), and two representing physical stress (physical demands and work conditions). To reduce data dimensionality, we used a principal component analysis (PCA) and a varimax rotation. It resulted in three constructs with eigenvalues > 1 that represented “job complexity,” “psychological stress,” and “physical stress” (Table 2).

**TABLE 2:** Results of factor analysis with PCA on select occupational characteristics.

	Job complexity	Psychological stress	Physical stress
% variance explained	37%	19%	12%
**Factor loadings:**			
Task variability	0.729		
Processing speed	0.754		
Problem solving	0.686		
Skill variety	0.514		
Sense of achievement	0.893		
Specialization	0.743		
Complexity	0.548	0.567	
Workload		0.662	
Interpersonal conflict		0.807	
Work conditions			−0.787
Physical demands			0.905

### Cognitive Function

To measure the latent constructs of reasoning, perceptual speed, episodic memory, and vocabulary knowledge we administered a well-validated Virginia Cognitive Aging Project cognitive battery (Salthouse and Ferrer-Caja, 2003; Salthouse, 2004, 2010) consisting of 16 computer-based and pen-and-pencil tasks. To obtain components representing the four cognitive constructs and to confirm the validity of task structure, we performed a PCA with varimax rotation, with missing values replaced by the sample mean (Burzynska et al., 2015). Data were within 3SD and normally distributed.

### General Stress and Leisure-Time Physical Activity

As an attempt to tease apart occupational physical demands from leisure physical activity, we used a well-validated 10-item Physical Activity Scale for the Elderly (PASE) (Washburn et al., 1993). We summed the weighted PASE scores related to leisure physical activities walking outside, light, moderate and strenuous sport/recreational activities, muscle strength/endurance exercises, light and heavy housework, home repairs, lawn work or yard care, outdoor gardening, and care for another person, omitting the score related to work or volunteering (Washburn et al., 1999).

To tease apart occupational from general psychological stress, we used the 10-item Perceived Stress Scale [PSS-10; (Cohen and Williamson, 1988)]. PSS-10 evaluates the degree to which individuals believe their life has been unpredictable, uncontrollable, and overloaded during the previous month on a 5-point Likert scale, with total score ranging from 0 to 40 (higher scores indicating higher levels of stress).

### Statistical Analyses

We used a linear hierarchical regression model (SPSS v. 26), with total hippocampal volume or the four cognitive constructs as dependent variables and the three occupational measures and other covariates as independent variables. R^2^ change statistics were used to determine whether occupational characteristics explained a significant amount of variance in the dependent variable, after controlling for the covariates. The covariates were age, gender, eTIV (for the model with hippocampal volume), and socioeconomic factors: years of completed formal education, household income, and job title (dummy coded, with homemaker as reference), “years between MRI/cog and occupational data,” “years in the occupation,” and current employment status. The same analyses were performed with adding either the general stress or leisure physical activity variables as covariates. The assumptions of linearity, normality of distributed errors, and uncorrelated errors were tested and fulfilled by the regression models.

## Results

### Occupational Characteristics, Hippocampal Volume, and Cognition

Only physical stress predicted hippocampal volume in the fully adjusted model [*R*^2^ = 0.419, *R*^2^_change_ = 0.074, *F*(3,74) = 3.1, *p* = 0.031; Table 3].

**TABLE 3:** Occupational correlates of hippocampal volume.

Independent variable	Std. beta	*t*	*p*	VIF
Age	–0.466	–4.6	<0.001	1.3
Gender	–0.115	–1.1	0.275	1.4
eTIV	0.204	1.7	0.085	1.7
Education	–0.035	–0.317	0.752	1.6
Income	–0.056	–0.557	0.579	1.3
Officials	0.137	0.339	0.736	20.1
Professional	0.071	0.0168	0.867	22.9
Technician	–0.005	–0.025	0.980	4.3
Sales	0.129	0.0541	0.590	7.2
Clerical	0.086	0.0256	0.799	14.2
Skilled	0.094	0.515	0.608	4.2
Semiskilled	0.214	0.796	0.429	9.2
Unskilled	0.127	0.967	0.337	2.2
Service	0.030	0.137	0.891	6.1
Employment status	–0.10	–0.091	0.928	1.5
Years in occupation	0.036	0.0361	0.719	1.2
Years MRI/cog − occupation	–0.072	–0.728	0.469	1.2
Job complexity	0.017	0.152	0.879	1.6
Occupational psychological stress	0.095	0.957	0.342	1.2
Occupational physical stress	–0.315	–2.9	**0.005**	1.5

**VIF, variance inflation factor. eTIV, estimated total intracranial volume*. Bold values p < 0.05.*

Next, we explored the associations between occupational characteristics and the four cognitive constructs. Only physical stress predicted a significant amount of variance in memory [*R*^2^ = 0.400, *R*^2^_change_ = 0.082, *F*(3,78) = 3.5, *p* = 0.018; Table 4].

**TABLE 4:** Occupational correlates of memory.

Independent variable	Std. beta	*t*	*p*	VIF
Age	–0.039	–0.390	0.697	1.3
Gender	0.476	4.8	<0.001	1.3
Education	–0.085	–0.769	0.444	1.6
Income	0.027	0.256	0.798	1.4
Officials	–0.389	–0.969	0.336	20.1
Professional	–0.439	–1.0	0.302	23.2
Technician	0.086	0.474	0.637	4.3
Sales	–0.186	–0.791	0.432	7.2
Clerical	–0.276	–0.834	0.407	14.3
Skilled	–0.132	–0.738	0.463	4.2
Semiskilled	–0.093	–0.321	0.759	11.0
Unskilled	–0.031	–0.241	0.810	2.2
Service	–0.072	–0.308	0.759	7.0
Employment status	–0.098	–0.959	0.340	1.3
Years in occupation	–0.076	–0.764	0.447	1.3
Years MRI/cog − occupation	0.043	0.446	0.657	1.2
Job complexity	0.088	0.810	0.420	1.5
Occupational psychological stress	0.184	1.9	0.058	1.2
Occupational physical stress	–0.292	–2.8	**0.007**	1.5

**VIF, variance inflation factor*. Bold values p < 0.05.*

### Physical Demands at Work, Leisure Physical Activity, and General Stress

The physical stress construct referred to both physical demands and work conditions. *Post hoc* two-tailed bivariate correlations indicated that “physical demands” correlated with hippocampal volume (*r* = −0.305, *p* = 0.003, *n* = 96) and memory (*r* = −0.247, *p* = 0.014, *n* = 99) but not with “work conditions.” Thus, we investigated whether occupational physical demands were related to hippocampal volume or memory beyond leisure physical activity or general stress.

After controlling additionally for leisure physical activity, physical stress remained associated with hippocampal volume (β_physical_stress_ = −0.367, *t* = −3.4, *p* = 0.001) and leisure physical activity was also positively associated with hippocampal volume (β_leisure physical activity_ = 0.195, *t* = 2.01, *p* = 0.048). The result was similar when controlling for general stress, with only physical stress being associated with hippocampal volume (β_physical_stress_ = −0.317, *t* = −2.9, *p* = 0.005). General stress was not related to hippocampal volume.

After controlling for leisure physical activity, the association of physical stress at work with memory remained significant (β_physical_stress_ = −0.287, *t* = −2.6, *p* = 0.011). After controlling for general stress, both physical stress (β_physical_stress_ = −0.295, *t* = −2.8, *p* = 0.007) and psychological stress at work (β_psychological_stress_ = 0.203, *t* = 2.1, *p* = 0.040) were associated with memory. General stress and leisure physical activity were not related to memory.

## Discussion

Our results indicate that physical stress, comprising physical demands and work conditions, was associated with smaller hippocampal volume and poorer memory performance. These associations were independent of age, gender, brain size, socioeconomic factors (education, income, and job title), duration of the job, time passed between MRI/cognitive and occupational data collection, employment status, general stress, and leisure physical activity. In addition, there was a weak positive association of leisure physical activity with hippocampal volume, and of occupational psychological stress with memory.

### Occupational Physical Stress and Hippocampal Volume

The main result of this study was a negative association between physical stress at work and hippocampal volume. In general, our results give support to the brain maintenance model of cognitive aging (Nyberg, 2017), where presence of a stressor may deplete brain health and memory, and the absence of the stressor may be protective. Importantly, *post hoc* correlations indicated that this association was driven by the “physical demand” aspect of occupational physical stress (i.e., need for muscular strength, endurance, and physical effort), with a lesser role for working conditions (e.g., environmental hazards). However, it is important to note that a very small portion of our sample worked in occupations that are defined as physical labor; only one person identified as an unskilled laborer and six as service workers. Therefore, our findings likely refer to one’s subjective or job-specific physical demand, beyond the official job title or a general job definition.

The negative physical demand-hippocampal volume association is an intriguing result, given that aerobic fitness, aerobic exercise, as well as resistance training have been shown to increase hippocampal volume and improve cognitive performance in older adults (Erickson et al., 2011; Szabo et al., 2011; Best et al., 2015; Ten Brinke et al., 2015). However, we found that controlling for leisure physical activity did not attenuate this negative association, although leisure physical activity showed the expected positive association with hippocampal volume. This suggests that leisure physical activity and physical demands at work may have independent and opposing associations with hippocampal structure and warrant replication and further study in larger samples.

Research on both animals and humans has demonstrated the negative effects of stress on the function and structure of the hippocampus (McEwen, 2006). However, to our knowledge, our results are novel in that we showed negative associations of (a) occupational and (b) subjective physical stress with hippocampal volume. Physical restraint or forced swimming has been used to induce physical stress in mice, resulting in increased oxidative stress (Nagata et al., 2009) or reduced serotonin receptor density in the hippocampus (Choi et al., 2014). With no doubt, more research is needed to understand the effects of occupational physical stress on the structure and function of the human hippocampus. Importantly, our data indicated that the occupational physical stress-hippocampus association was independent of general stress, suggesting that this relationship may be specific to occupational experiences.

Finally, our data indicated that the association between occupational physical stress and hippocampal volume was independent of socioeconomic factors, including early-life education, the related subsequent occupational attainment (i.e., job title), and income. This is in line with earlier studies linking occupational experiences to cognitive outcomes in older age, suggesting that the recency of occupational experiences may take precedence in predicting neural and cognitive outcomes in older age (Dartigues et al., 1992a, b; Frisoni et al., 1993).

### Occupational Characteristics and Cognition

We found that greater occupational physical stress was related to poorer episodic memory, independent of demographic and socioeconomic covariates. This is consistent with recent studies showing that older adults who reported high physical work strain showed declines in memory and general cognition (Gow et al., 2014; Sindi et al., 2017; Dong et al., 2018), controlling for sex, education and social class. These studies resembled ours in the subjective assessment of physical demands, physical hazards, and risk of injury at work, but assessed cognitive ability using single tasks or crude composite scores. Therefore, our study extends these earlier findings by a using a broader construct of memory.

Interestingly, our findings also converge with studies that used occupational category as a proxy of physical stress at work. For instance, an epidemiological study in France found that older farmers, service employees, or blue-collar workers had higher risk of memory (Dartigues et al., 1992b) or general cognitive decline (Dartigues et al., 1992a) than those working in professional/managerial occupations, controlling for age, sex, and education. A similar association was found among older adults in Italy, after adjustment for age, education, and financial dissatisfaction (Frisoni et al., 1993). Thus, the association between physical stress at work and poorer cognitive abilities in older age, independent of socioeconomic status, seems to hold across different countries and operationalizations of physical stress.

In addition, we found a weak but positive association between occupational psychological stress (i.e., workload and intrapersonal conflict) and memory when controlling for general stress. This finding is an important addition to the mixed results linking work-related psychological stress to cognition in later life: one study reported that greater work strain predicted poorer memory performance and greater memory decline 15–21 years later (Agbenyikey et al., 2015). Another study found that midlife work-related stress predicted poorer global cognition and processing speed 25 years later, but was unrelated to episodic memory, executive functioning, and verbal fluency (Sindi et al., 2017). Similarly, adults who worked in jobs with high psychological demands or strain had greater 11-year declines in MMSE scores that those in low strain jobs, with no difference in verbal memory (Dong et al., 2018). These discrepancies may stem from different operationalizations of cognitive function, work-related stress, and study timelines. In sum, as research in both animals and humans shows that stress can facilitate memory and learning under some conditions (e.g., Joëls et al., 2006) our preliminary findings warrant further research.

Finally, we did not find positive associations between occupational complexity, hippocampal size and cognition. This resembles findings by Oltmanns et al. (2017) and Kaup et al. (2018), but not Suo et al. (2012), suggesting that different operationalizations of occupational stimulation (e.g., novelty at work, managerial experience, complexity with data or people, or subjective variety of tasks or information processing) may lead to different outcomes.

### Limitations and Future Directions

The main limitation of our study was the retrospective collection of occupational data, which occurred several years after the MRI and cognitive measurements. Although controlling for this time lag, job duration, or employment status did not change the results, we do not know whether our participants referred to their current or past job, how long ago they retired from the job, and whether it was their main lifetime occupation, a later post-retirement, or a bridge job. Furthermore, although all our participants scored high on the MMSE at the time of MRI and cognitive data collection, it remains unknown whether and to what extent their responses to occupational questionnaire were affected by age-related declines in memory (Park and Festini, 2017) or the positivity effect (Mather and Carstensen, 2005). Future studies could consider combining occupational surveys with 24-h recalls (Matthews et al., 2018) or real-time assessments such as the Ecological Momentary Assessment (Shiffman et al., 2008) to gauge the nature of job tasks, their intellectual and physical challenge, objective stress, and emotional reactivity with lesser recall bias and greater ecological validity. Next, our cross-sectional approach does not allow for any causal interpretations. Thus, the observed associations are equally likely to be bidirectional (i.e., one’s memory ability or brain health could affect one’s occupational choices or opportunities) or driven by a third variable that was not captured in our study but could affect both hippocampal volume, memory, and perception of work environment, for example, quality of sleep, metabolic disorders such as obesity and diabetes, chronic pain and inflammation, and depression (Fotuhi et al., 2012; Mutso et al., 2012). However, our approach is the next best way to establish the relationships between occupational exposures, brain and cognitive health, given it is not possible to experimentally manipulate long-term occupational exposures in a randomized design. Future observational longitudinal studies may help establish time-ordered associations. Finally, our promising results should be extended to other brain regions and metrics of brain health, and possible interactions of occupational stress and stimulation on brain and cognition need to be explored.

## Conclusion

Using validated questionnaires of subjective work characteristics, we found a negative association between occupational physical demands, hippocampal volume, and memory in cognitively healthy older adults. The observed associations were independent of early-life education and socioeconomic factors, which highlights the importance of considering occupational experiences in understanding individual trajectories of cognitive and brain aging. Our findings suggest that future interventions aimed at maintaining hippocampal and cognitive health may need to target both workplace and leisure activities.

## Data Availability Statement

The raw data supporting the conclusions of this article will be made available by the authors, without undue reservation, to any qualified researcher.

## Ethics Statement

The studies involving human participants were reviewed and approved by University of Illinois institutional review board. The patients/participants provided their written informed consent to participate in this study and the study was performed in accordance with the 1964 Declaration of Helsinki.

## Author Contributions

AB, DG, AK, and EM conceived and designed the occupational data collection. MV, AK, and EM conceived and designed the MRI and cognitive data collection. AB, JF, ES, and NG collected and preprocessed the data. AB and DG performed the statistical analysis. AB wrote the first draft of the manuscript. All authors contributed to manuscript revision, read and approved the submitted version.

## Conflict of Interest

The authors declare that the research was conducted in the absence of any commercial or financial relationships that could be construed as a potential conflict of interest.

## Footnotes

1 http://surfer.nmr.mgh.harvard.edu/
