# Supplementary material for: Occupational Physical Stress Is Negatively Associated With Hippocampal Volume and Memory in Older Adults
Source: Front Hum Neurosci. 2020 Jul 15;14:266. doi: 10.3389/fnhum.2020.00266 (PMC7381137; doi:10.3389/fnhum.2020.00266)
Supplement: Supplementary file 1 [file Table_1.pdf]

Burzynska et al. 2020 “Occupational physical stress is negatively associated with hippocampal volume and memory in older adults”

Supplementary Material:
Occupational experience questionnaire

Work Design Questionnaire (WDQ; Morgeson and Humphrey, 2006), by dimension:

Autonomy
Work Scheduling Autonomy
1. The job allows me to make my own decisions about how to schedule my work.
2. The job allows me to decide on the order in which things are done on the job.
3. The job allows me to plan how I do my work.

Decision-Making Autonomy
1. The job gives me a chance to use my personal initiative or judgment in carrying out the work.
2. The job allows me to make a lot of decisions on my own.
3. The job provides me with significant autonomy in making decisions.

Work Methods Autonomy
1. The job allows me to make decisions about what methods I use to complete my work.
2. The job gives me considerable opportunity for independence and freedom in how I do the work.
3. The job allows me to decide on my own how to go about doing my work.

Task Variety
1. The job involves a great deal of task variety.
2. The job involves doing a number of different things.
3. The job requires the performance of a wide range of tasks.
4. The job involves performing a variety of tasks.

Task Significance
1. The results of my work are likely to significantly affect the lives of other people.
2. The job itself is very significant and important in the broader scheme of things.
3. The job has a large impact on people outside the organization.
4. The work performed on the job has a significant impact on people outside the organization.

Task Identity
1. The job involves completing a piece of work that has an obvious beginning and end.
2. The job is arranged so that I can do an entire piece of work from beginning to end.
3. The job provides me the chance to completely finish the pieces of work I begin.
4. The job allows me to complete work I start.

Feedback From Job
1. The work activities themselves provide direct and clear information about the effectiveness (e.g., quality and quantity) of my job performance.
2. The job itself provides feedback on my performance.
3. The job itself provides me with information about my performance.

Job Complexity
1. The job requires that I only do one task or activity at a time (reverse scored).

2. The tasks on the job are simple and uncomplicated (reverse scored).
3. The job comprises relatively uncomplicated tasks (reverse scored).
4. The job involves performing relatively simple tasks (reverse scored).

Information Processing
1. The job requires me to monitor a great deal of information.
2. The job requires that I engage in a large amount of thinking.
3. The job requires me to keep track of more than one thing at a time.
4. The job requires me to analyze a lot of information.

Problem Solving
1. The job involves solving problems that have no obvious correct answer.
2. The job requires me to be creative.
3. The job often involves dealing with problems that I have not met before.
4. The job requires unique ideas or solutions to problems.

Skill Variety
1. The job requires a variety of skills.
2. The job requires me to utilize a variety of different skills in order to complete the work.
3. The job requires me to use a number of complex or high-level skills.
4. The job requires the use of a number of skills.

Specialization
1. The job is highly specialized in terms of purpose, tasks, or activities.
2. The tools, procedures, materials, and so forth used on this job are highly specialized in terms of purpose.
3. The job requires very specialized knowledge and skills.
4. The job requires a depth of knowledge and expertise.

Social Support
1. I have the opportunity to develop close friendships in my job.
2. I have the chance in my job to get to know other people.
3. I have the opportunity to meet with others in my work.
4. My supervisor is concerned about the welfare of the people that work for him/her.
5. People I work with take a personal interest in me.
6. People I work with are friendly.

Interdependence
Initiated Interdependence
1. The job requires me to accomplish my job before others complete their job.
2. Other jobs depend directly on my job.
3. Unless my job gets done, other jobs cannot be completed.

Received Interdependence
1. The job activities are greatly affected by the work of other people.
2. The job depends on the work of many different people for its completion.
3. My job cannot be done unless others do their work.

Burzynska et al. 2020 “Occupational physical stress is negatively associated with hippocampal volume and memory in older adults”

Interaction Outside Organization
1. The job requires spending a great deal of time with people outside my organization.
2. The job involves interaction with people who are not members of my organization.
3. On the job, I frequently communicate with people who do not work for the same organization as I do.
4. The job involves a great deal of interaction with people outside my organization.

Feedback From Others
1. I receive a great deal of information from my manager and coworkers about my job performance.
2. Other people in the organization, such as managers and coworkers, provide information about the effectiveness (e.g., quality and quantity) of my job performance.
3. I receive feedback on my performance from other people in my organization (such as my manager or coworkers).

Ergonomics
1. The seating arrangements on the job are adequate (e.g., ample opportunities to sit, comfortable chairs, good postural support).
2. The work place allows for all size differences between people in terms of clearance, reach, eye height, leg room, etc.
3. The job involves excessive reaching (reverse scored).

Physical Demands
1. The job requires a great deal of muscular endurance.
2. The job requires a great deal of muscular strength.
3. The job requires a lot of physical effort.

Work Conditions
1. The work place is free from excessive noise.
2. The climate at the work place is comfortable in terms of temperature and humidity.
3. The job has a low risk of accident.
4. The job takes place in an environment free from health hazards (e.g., chemicals, fumes, etc.).
5. The job occurs in a clean environment.

Equipment Use
1. The job involves the use of a variety of different equipment.
2. The job involves the use of complex equipment or technology.
3. A lot of time was required to learn the equipment used on the job.

Interpersonal Conflict at Work Scale (Spector & Jex, 1988)

1. How often did you get into arguments with others at work?
2. How often did other people yell at you at work?
3. How often were people rude to you at work?
4. How often did other people do nasty things to you at work?

Burzynska et al. 2020 "Occupational physical stress is negatively associated with hippocampal volume and memory in older adults"

Quantitative Workload Inventory (Spector & Jex, 1988)

1. How often did your job require you to work very fast?
2. How often did your job require you to work very hard?
3. How often did your job leave you with little time to get things done?
4. How often was there a great deal to be done?
5. How often did you have more work than you could do well?
